# The relationship between glucose intolerance status and risk of hospitalization during two decades of follow-up: Tehran lipid and glucose study

**DOI:** 10.1080/07853890.2022.2143552

**Published:** 2022-11-16

**Authors:** Rahele Rasooli, Azra Ramezankhani, Davood Khalili, Maryam Tohidi, Mitra Hasheminia, Fereidoun Azizi, Farzad Hadaegh

**Affiliations:** a Prevention of Metabolic Disorders Research Center, Research Institute for Endocrine Sciences, Shahid Beheshti University of Medical Sciences, Tehran, Iran; b Endocrine Research Center, Research Institute for Endocrine Sciences, Shahid Beheshti University of Medical Sciences, Tehran, Iran

**Keywords:** Glucose intolerance, pre-diabetes, diabetes, hospitalization

## Abstract

**Objective:**

To assess the relationship between glucose intolerance statuses at baseline defined as normal glucose tolerance (NGT), pre-diabetes, newly diagnosed (NDM) and known diabetes mellitus (KDM) and all-cause hospitalization among Iranian men and women during 20 years of follow-up.

**Research design and methods:**

This study included 8,014 individuals (3,836 men) ≥30 years from the cohort of Tehran Lipid and Glucose Study. Incidence rate ratios (IRRs) and (95% confidence interval (95% CI) for three groups of pre-diabetes, NDM and KDM was estimated using the Negative Binomial regression model, considering NGT group as reference group. Regression models were adjusted for age, body mass index, hypertension, chronic kidney disease, and cardiovascular disease (CVD).

**Results:**

Among men, compared with NGT group, those with pre-diabetes, NDM and KDM had higher incidence rate for hospitalization, with IRRs (95% CI) of 1.08 (0.96–1.20), 1.38 (1.20–1.57) and 1.96 (1.66–2.26), respectively, after adjusting for confounders. The corresponding values were 1.07 (0.96–1.17), 1.40 (1.21–1.59) and 2.07 (1.72–2.42) for women. Men with diabetes, generally had a higher rate of hospitalization for CVD rather than their female counterparts (IRRs: 1.46; 1.17–1.74). In patients with diabetes, the most common causes of hospitalization were macrovascular complications (i.e. coronary heart disease and stroke). Moreover, among the individuals with diabetes, those with poor glycaemic control (fasting plasma glucose (FPG) >10 mmol/l) had 39% higher rate of hospitalization than those with fair glycaemic control (FPG <10 mmol/l) (1.39; 1.12–1.65), adjusted for confounders.

**Conclusion:**

Pre-diabetes, NDM, and KDM were associated with increased hospitalization rates during long-term follow-up. Interventions such as lifestyle modification or pharmacological therapies aiming to slow down the pre-diabetes and fair control of diabetes might potentially decrease the rate of hospitalization.

**Key messages**
- NDM and KDM status both increased rate of all-cause hospitalization.
- CVD and T2DM complication were the most common cause of hospitalization among patients with diabetes.
- Hospitalization due to recurrent CHD was significantly higher in men with diabetes than their female counterparts.

## Introduction

Type 2 diabetes mellitus (T2DM) as a chronic disease affecting 537 million adults (aged 20–79 years), worldwide. About 6.7 million deaths were attributed to diabetes in 2021, and by 2045, there would be 783 million people with diabetes [1]. A national study in Iran conducted in 2016 found that prevalence of diabetes was about 10.01 and 11.55% in men and women, respectively [2]. Moreover, each year more than one percent of Iranian adults develop T2DM [3]. It has been projected that in Iran the number of people with diabetes to increase by 91% by 2035 [4]. Despite increasing prevalence and incidence of diabetes in Iran, a trend analysis in 2005–2011 found that the awareness about diabetes improved in the country, and prevalence of undiagnosed diabetes decreased from 45.7% to 24.7% [5].

Although T2DM diagnosis and treatment have improved, only 50% of patients reach the targeted glycaemic indices [6]. By failure to achieve therapeutic goals, micro and macrovascular complications are increasing as the duration of the disease increases [7], which can cause more hospitalizations. We found that the pre-diabetes status among the Tehran population was associated with incident hypertension, chronic kidney disease (CKD), and cardiovascular disease (CVD) [8], conditions potentially accompanied by more hospitalization. In Iran, the total cost of hospitalization in a person with T2DM was 80 USD per year in 2009, which resulted in 194 million USD for the entire population [9].

Several studies have investigated the relation between pre-diabetes/diabetes and risk of hospitalization. However, most of these studies have investigated the relationship only for first hospitalization [10, 11]. Only few studies have addressed the impact of pre-diabetes/diabetes on total number of hospitalizations during study period [12], which better reflects the cumulative burden of increased hospitalization associated with this condition. Therefore, the current study was designed to assess the relationship between pre-diabetes and T2DM and total number of hospitalizations for any cause in adult men and women during 20 years follow up among participants of the Tehran lipid and glucose study (TLGS).

## Research design and methods

### Study population

The TLGS is a population-based cohort study conducted in 1999 in district 13 of Tehran. The original aim of the TLGS was to assess the prevalence and incidence of non-communicable diseases (NCD) and their risk factors. The study design, measurement, methods, and enrollment strategy have been explained in detail previously [13]. Briefly, a total of 15005 individuals aged ≥3 years were recruited in Phase 1 (1999–2001), and 3550 new subjects were included in Phase 2 (2002–2005). All participants were then followed up every three years since enrolment. In this study, we included 9558 participants ≥30 years from phase 1 (*n* = 7927) and phase 2 (*n* = 1631), as the baseline population, and excluded those with missing data on glucose tolerance status (*n* = 398), covariates (i.e. body mass index (BMI), smoking status, marital status, education level, hypercholesterolemia, hypertension, CKD and history of CVD (*n* = 267)), and finally, those without any follow-up data after baseline recruitment (*n* = 879), leaving us 8014 individuals (3638 men) (more than 80% of eligible participants) who were followed until the end of the study (20 March 2018) (Supplementary Figure 1). Study protocol was approved by the ethical committee of the Research Institute for Endocrine Sciences of Shahid Beheshti University of Medical Sciences, Tehran, Iran, and written informed consent was obtained from the study participants.

**Figure 1.**
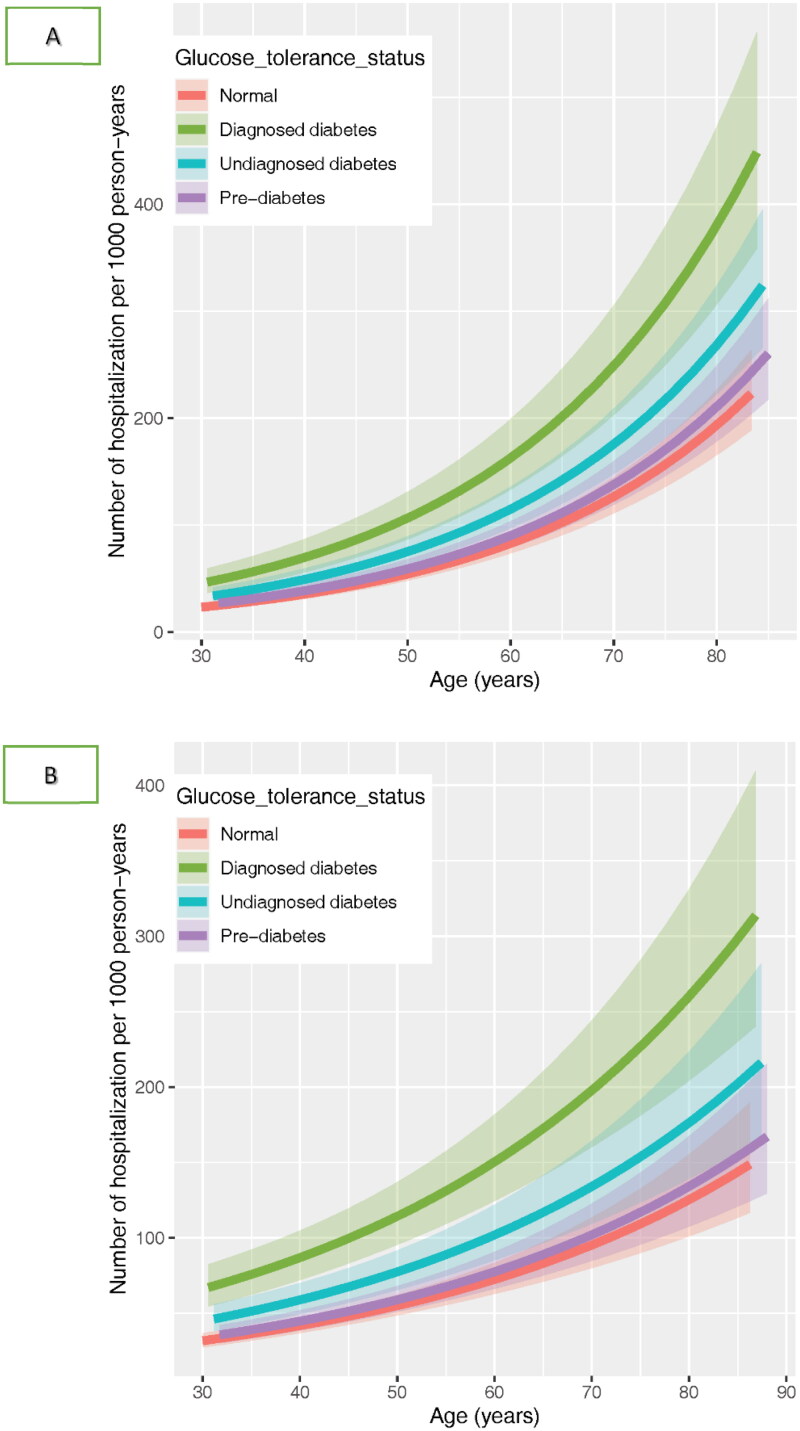
Prediction based on multivariable adjusted negative binomial models over the age range in men (A) and women (B).

### Measurement

According to a standard protocol, trained physicians examined all participants at the TLGS clinic. Demographic information such as age, sex, marital status, education level, and smoking status was obtained using a validated questionnaire. Anthropometric measures, including weight and height were recorded using standard protocols, and BMI was calculated as weight in kilograms divided by height in square meters (kg/m^2^). Blood pressure (BP) was measured twice in a seated position after 15 min resting using a standard mercury sphygmomanometer. A blood sample was drawn between 7:00 and 9:00 AM from all study participants after 12–14 h of overnight fasting to measure biochemical measurements, including fasting plasma glucose (FPG), total cholesterol (TC), and triglyceride (TG). For the oral glucose tolerance test (OGTT), blood glucose was measured 2 h after oral administration of 82.5 g of glucose monohydrate solution (equivalent to 75 g anhydrous glucose) in subjects aged ≥20 years, excluding those taking glucose-lowering drugs medications.

The estimated glomerular filtration rate (e-GFR) was calculated based on serum creatinine using the CKD Epidemiology Collaboration (CKD-EPI) equation [14].

### Definition of terms

At baseline, known diabetes mellitus (KDM) was defined as a self-report of diabetes diagnosis by a physician or using glucose-lowering medication. Then, we categorized people without known diabetes into 3 categories based on their baseline laboratory data including FPG and 2-hour post-challenge glucose (2-hrPCG): (1) normal glucose tolerance (NGT) (FPG <5.6 mmol/l and 2-hrPCG <7.8 mmol/l); (2) pre-diabetes (5.6 mmol/l < FPG <7mmol/l or 7.8 mmol/*l* < 2-hrPCG <11.1 mmol/l), and (3) newly diagnosed T2DM (NDM) (FPG ≥7 mmol/l or 2-hrPCG ≥11.1 mmol/l). As we had no data on HbA1c, T2DM control in patients with KDM was categorized using the FPG values in to category of poor controlled (FPG ≥10 mmol/l, which was considered as a surrogate of HbA1C level ≥8%) and fair controlled (FPG <10 mmol/l, as a surrogate of HbA1C level of 7–8%) [15]. Marital status was divided into three categories: single, married, widowed/divorced. Educational level was categorized as: less than six years, 6–12 years, and more than 12 years. Smoking status was defined as current smoker, past-smoker, and never smoker. BMI was categorized into three groups: normal (<25 kg/m^2^), overweight (25–29.9 kg/m^2^), and obese (≥30 kg/m^2^). Hypertension was defined as systolic blood pressure (SBP) ≥140 mmHg or diastolic blood pressure (DBP) ≥90 mmHg or using antihypertensive medications [16]. Hypercholesterolemia was defined as having TC ≥5.1 mmol/l or using the lipid-lowering drugs [17]. CKD was defined as having e-GFR <60 ml/min/1.73 m^2^ [18].

### Assessment of outcomes

Detailed assessment for outcomes in TLGS has been addressed elsewhere [19]. In brief, TLGS participants were followed annually by telephone call to them or their family. They were asked for any medical event leading to hospitalization during the past year. The response rate in annual telephone call follow-ups was about 80–90% [19]. In case of positive responses, a trained physician collected related data through home visits or hospital records. In the case of mortality, data were collected from hospital records or health certificates. Data were reviewed by an outcome committee consisting of an internist, endocrinologist, cardiologist, epidemiologist, pathologist, and physician who collected outcome data. The primary cause for hospitalization was defined according to ICD-10 criteria (Supplementary Table 1).

The main outcome in this study was the total count of all-cause hospitalization during follow-up period. We excluded the hospitalizations for the caesarean section, normal vaginal delivery, cosmetic surgeries, and laser therapy for diabetic retinopathies. The primary cause of hospitalizations was categorized into nine groups: coronary heart disease (CHD), stroke, infectious disease, respiratory disease, T2DM complications, hypertension complications, neoplasm, traumas, and others (Supplementary Table 1).

### Statistical analysis

Baseline characteristics of the participants are presented by sex and glucose tolerance status and were compared using one-way analysis of variance (ANOVA) and chi-squared tests for continuous and categorical variables, respectively. We further compared baseline characteristics between included and non-included individuals using t-test and chi-squared tests. Non-included individuals were those with missing data on glucose tolerance status and other covariates at baseline and individuals without any follow-up data.

Total numbers of all-cause and cause-specific hospitalizations are presented by glucose tolerance status in men and women, separately. For overall population, the crude rates of all-cause and cause-specific hospitalization per 1000 person-years with 95% confidence interval (95% CI) was calculated by glucose tolerance status. The follow-up time was defined as the interval between date of study enrollment and date of last follow-up measured in years.

In this study, we considered the total number of hospitalization as a count variable and fitted a negative binomial (NB) regression to investigate relation between total number of all-cause hospitalizations and glucose tolerance status at baseline, separately in men and women. The NB regression preferred over the Poisson models, because likelihood ratio test of α = 0 was significant with *p* < 0.001. Since the participants did not have the equal follow-up time (due to loss to follow-up or death during study period), we included the follow-up time as an offset variable in NB models to account for the different follow-up times. In model 1, we adjusted for age. Model 2 was further adjusted for educational level, marital status, and smoking status. In model 3, we included all variables in model 2 plus BMI category, hypertension, hypercholesterolemia, CKD, and history of CVD. The incidence rate ratios (IRRs) are reported for NB models and 95% CI for the IRRs were computed based on robust standard errors (SE). To estimate adjusted rates of hospitalization per 1000 person-years, we used the fitted NB models (model 3). In fact, adjusted rates of hospitalization are the outcome predicted by a fitted model, where all independent variables are held at specified values. It should be noted that we can consider different combination of values for the predictor variables to estimate the adjusted rates. We estimated adjusted rates by setting the continuous variables to the mean and categorical variables to the reference category which were the most frequent category (mode). Continuous covariates included age and categorical covariates included smoking status (current, past, never [reference]), education status (>12 years, 6–12 years, <6 years [reference]), marital status (single, widowed/divorced, married [reference]), BMI category (obese, overweight, normal [reference], hypertension (yes, no [reference], hypercholesterolemia (yes, no [reference], CKD (yes, no [reference] and history of CVD (yes, no [reference].

We also plotted the adjusted rates per 1000 person-years over the age range, using the multi-variable NB model (model 3). The values of the independent variables were similar to those mentioned above.

In a sensitivity analysis, we also fitted the age-adjusted zero-inflated negative binomial (ZINB) regression model because about 45% of men and women were never hospitalized during follow-up (zero counts). The age-adjusted ZINB regression models were then used to calculate age-adjusted hospitalization rates per 1000 person-years (95% CI) when age was set to mean value.

We additionally performed a sensitivity analysis among persons with KDM, and examined the effect of glycaemic control status on the hospitalization rate in the multi-variable NB models.

Since a considerable number of participants (40–60%) did not have data on 2-hr PCG during different follow-up examinations, we could not accurately categorize all participants for glucose tolerance status, and therefore, we were unable to perform time-varying analyses. Instead, like the Atherosclerosis Risk in Communities (ARIC) study [12] and as a sensitivity analysis, we limited follow-up time to 5 and 10 years after baseline and repeated all main analysis to explore the possible impact of changing from NGT to pre-diabetes/diabetes and changing from pre-diabetes to diabetes on future risk of all cause hospitalization.

The Akaike information criterion (AIC) was used to compare the goodness of fit poison and NB model. To compare model fitting between age adjusted NB and ZINB regression models we used the Vuong test [20].

Analyses were performed using IBM SPSS Statistics version 20 (IBM Corp) and R version 4.1.2. In all analyses, two-sided P-values <0.05 were considered statistically significant.

## Results

Supplementary Table 2 shows the baseline characteristics of included and non-included individuals. Compared with the included, the non-included persons were older and had a lower mean BMI and e-GFR. They were more likely than included subjects to be smoker. Non-included individuals also were more likely to have prevalent CVD and CKD than included individuals.

The study population included 3638 men and 4373 women, with the mean age of 53.1 and 50.6 years, respectively. Among men, 63.5% were NGT, 23% had pre-diabetes, 9.4% had NDM, and 4.1% had KDM. Men in NGT group, were significantly younger and had lower BMI, SBP, DBP, FPG, TC, and TG, but higher e-GFR, compared with other groups. Also, they were less likely to be married, have a history of CVD, CKD, hypertension, and hypercholesterolemia, but more likely to smoke than men in other groups (Table 1).

**Table 1.** Baseline characteristics of men (*n* = 3638) by glucose tolerance status: the TLGS study, 1999–2018.

	Normal (*n* = 2311)	Pre-diabetes (*n* = 836)	Newly diagnosed diabetes (*n* = 343)	Known diabetes (*n* = 148)	*p* Value
**Continuous variables**					
Age (years)	45.5 (12.4)	51.2 (12.8)	55.7 (11.7)	60.1 (10.7)	<0.001
BMI (kg/m^2^)	25.7 (3.8)	27.0 (3.9)	28.1 (3.8)	26.5 (3.7)	<0.001
SBP (mmHg)	118.0 (17.5)	126.5 (18.8)	134.2 (22.9)	132.2 (23.4)	<0.001
DBP (mmHg)	77.0 (10.8)	80.5 (11.6)	82.3 (12.5)	79.9 (11.3)	<0.001
FPG (mmol/L)	4.8 (0.4)	5.6 (0.5)	8.4 (3.1)	9.5 (3.2)	<0.001
TC (mmol/L)	5.2 (1.1)	5.5 (1.1)	5.7 (1.2)	5.4 (1.2)	<0.001
TG (mmol/L)	1.9 (1.2)	2.4 (1.7)	3.0 (2.3)	2.4 (1.6)	<0.001
e-GFR (mL/min per 1.73 m^2^)	76.3 (13.4)	70.8 (13.6)	68.8 (13.7)	66.7 (13.2)	<0.001
**Categorical variables**					
Smoking (%)					
Never	1179 (51.0)	450 (53.8)	177 (51.6)	88 (59.5)	<0.001
Past	347 (15.0)	159 (19.0)	78 (22.7)	31 (20.9)	
Current	785 (34.0)	227 (27.2)	88 (25.7)	29 (19.6)	
Marital status (%)					
Single	122 (2.4)	20 (2.4)	3 (0.9)	0 (0.0)	<0.001
Married	2170 (93.9)	807 (96.5)	335 (97.7)	145 (98.0)	
Widowed/divorced	19 (0.8)	9 (1.1)	5 (1.5)	3 (2.0)	
Educational level (%)					
<6 years	574 (24.8)	314 (37.6)	152 (44.3)	79 (53.4)	<0.001
6–12 years	1281 (55.4)	404 (48.3)	147 (42.9)	52 (35.1)	
≥12 years	456 (19.7)	118 (14.1)	44 (12.8)	17 (11.5)	
Prevalent CVD (yes)	108 (4.7)	66 (7.9)	47 (13.7)	36 (24.3)	<0.001
Hypertension (yes)	404 (17.5)	280 (33.5)	157 (45.8)	62 (41.9)	<0.001
CKD (yes)	247 (10.7)	163 (19.5)	88 (25.7)	51 (34.5)	<0.001
Hypercholesterolemia (yes)	1188 (51.4)	519 (62.1)	237 (69.1)	90 (60.8)	<0.001
BMI status (%)					
Normal	1018 (44.1)	242 (28.9)	69 (20.1)	51 (34.5)	<0.001
Overweight	999 (43.2)	427 (51.1)	171 (49.9)	72 (48.6)	
Obese	294 (12.7)	167 (20.0)	103 (30.0)	25 (16.9)	
All cause death until end of study	260 (11.3)	142 (17.0)	106 (30.9)	82 (55.4)	<0.001

Values are shown as mean (standard deviation) for continuous variables and frequency (percent) for categorical variables.

BMI: body mass index; SBP: systolic blood pressure; DBP: diastolic blood pressure; FPG: fasting plasma glucose;

2h-PLPG: 2-h post load plasma glucose; TC: total cholesterol; TG: triglycerides; e-GFR: estimated glomerular filtration rate; CKD: chronic kidney disease; CVD: cardiovascular diseases.

Among women, 61.3% were NGT, 23.4% had pre-diabetes, 9.3% had NDM, and 6% had KDM. Women in NGT group, were younger, had lower BMI, SBP, DBP, FPG, TC, TG, but higher e-GFR, than women in other groups. They also smoked more, had higher education level, and were more likely to be married, but were less likely to have prevalent CVD, CKD, hypertension, and hypercholesterolemia (Table 2).

**Table 2.** Baseline characteristics of women (*n* = 4376) by glucose tolerance status: the TLGS study, 1999–2018.

	Normal (*n* = 2683)	Pre-diabetes (*n* = 1025)	Newly diagnosed diabetes (*n* = 407)	Known diabetes (*n* = 261)	*p* Value
**Continuous variables**					
Age (years)	43.6 (10.9)	49.4 (11.1)	52.5 (10.3)	56.9 (9.6)	<0.001
BMI (kg/m2)	27.8 (4.5)	29.8 (4.9)	30.2 (4.7)	29.2 (5.1)	<0.001
SBP (mmHg)	116.4 (17.8)	126.9 (20.2)	134.8 (22.3)	136.6 (23.2)	<0.001
DBP (mmHg)	76.9 (10.3)	81.5 (10.6)	83.5 (10.9)	81.9 (11.4)	<0.001
FPG (mmol/L)	4.8 (0.3)	5.5 (0.5)	8.3 (3.1)	11.0 (3.6)	<0.001
TC (mmol/L)	5.4 (1.2)	5.9 (1.2)	6.3 (1.4)	6.3 (1.3)	<0.001
TG (mmol/L)	1.7 (0.9)	2.2 (1.2)	2.9 (2.0)	2.7 (1.6)	<0.001
e-GFR (mL/min per 1.73 m^2^)	72.3 (12.7)	68.1 (12.4)	65.9 (12.0)	62.6 (12.9)	<0.001
**Categorical variables**					
Smoking (%)					
Never	2486 (92.7)	966 (94.2)	376 (92.4)	234 (89.7)	<0.001
Past	49 (1.8)	27 (2.6)	14 (3.4)	14 (5.4)	
Current	148 (5.5)	32 (3.1)	17 (4.2)	13 (5.0)	
Marital status (%)					
Single	137 (5.1)	21 (2.0)	6 (1.5)	2 (0.8)	<0.001
Married	2274 (84.8)	863 (84.2)	312 (76.7)	192 (73.6)	
Widowed/divorced	272 (10.1)	141 (13.8)	89 (21.9)	67 (25.7)	
Educational level (%)					
<6 years	1037 (38.7)	581 (56.7)	280 (68.8)	203 (77.8)	<0.001
6–12	1386 (51.7)	395 (38.5)	120 (29.5)	53 (20.3)	
≥12	260 (9.7)	49 (4.8)	7 (1.7)	5 (1.9)	
Prevalent CVD (yes)	84 (3.1)	56 (5.5)	29 (7.1)	51 (19.5)	<0.001
Hypertension (yes)	495 (18.4)	400 (39.0)	205 (50.4)	157 (60.2)	<0.001
CKD (yes)	416 (15.5)	268 (26.1)	125 (30.7)	111 (42.5)	<0.001
Hypercholesterolemia (yes)	1511 (56.3)	745 (72.7)	330 (81.1)	223 (85.4)	<0.001
BMI status (%)					
Normal	746 (27.8)	153 (14.9)	51 (12.5)	50 (19.2)	<0.001
Overweight	1144 (42.6)	401 (39.1)	160 (39.3)	108 (41.4)	
Obese	793 (29.6)	471 (46.0)	196 (48.2)	103 (39.5)	
All cause death until end of study	133 (5.0)	105 (10.2)	65 (16.0)	102 (39.1)	<0.001

Values are shown as mean (standard deviation) for continuous variables and frequency (percent) for categorical variables.

BMI: body mass index; SBP: systolic blood pressure; DBP: diastolic blood pressure; FPG: fasting plasma glucose;

2h-PLPG: 2-h post load plasma glucose; TC: total cholesterol; TG: triglycerides; e-GFR: estimated glomerular filtration rate; CKD: chronic kidney disease; CVD: cardiovascular diseases.

By moving from NGT to the known T2DM group, the percentage of people with no admission (zero counts) decreased, in both gender. Also, the percentage of people with more than three hospitalizations was higher in the T2DM than other groups (Supplementary Figure 2).

Excluding un-defined causes, the most frequent causes of hospitalization in men with NDM and KDM were CHD, T2DM complications, stroke (Supplementary Table 3), and in their female counterparts, it was CHD, T2DM complications, and infections (Supplementary Table 4). In both genders, the pre-diabetes and NGT group, CHD, neoplasm, and traumas were the most frequent cause for hospitalization (Supplementary Tables 3 and 4).

In total population, the crude rates (95% CI) of all-cause hospitalization per 1000 person-years were 62.6 (59.9–65.3), 88.1 (82.5–93.8), 134.4 (122.8–146.1), and 223.3 223.3 (199.3–247.3) in NGT, pre-diabetes, NDM, and KDM groups, respectively (Supplementary Table 5).

The incident rate ratios (IRRs) of hospitalization from NB models have been shown in Table 3. In both genders, after adjustment for age (model 1), the hospitalization rate was higher in participants with pre-diabetes and patients with NDM or KDM, compared to the NGT group. After further adjustment for demographic variables, the hospitalization rate remained higher in three groups with pre-diabetes, NDM, and KDM than the NGT group (model 2). After further adjustment for other covariates, including BMI, hypertension, hypercholesterolemia, CKD, and prevalent CVD, the pre-diabetes group did not have a higher hospitalization rate than the NGT. However, in both genders, the NDM group had about 40%, and KDM had around a 2-fold higher hospitalization rate than NGT group (model 3) (Table 3).

**Table 3.** Estimates of incident rate ratios (IRRs) (95% CI) from negative binomial regression models in men and women, the TLGS study, 1999–2018.

	Diabetes status	Men	Women
**Model 1**	NGT	Reference	Reference
	Pre-diabetes	1.13 (1.01–1.26)	1.13 (1.02–1.24)
	NDM	1.57 (1.35–1.78)	1.52 (1.31–1.73)
	KDM	2.21 (1.85–2.57)	2.32 (1.94–2.71)
**Model 2**	NGT	Reference	Reference
	Pre-diabetes	1.14 (1.02–1.27)	1.12 (1.01–1.23)
	NDM	1.56 (1.35–1.76)	1.50 (1.29–1.71)
	KDM	2.21 (1.86–2.56)	2.30 (1.91–2.69)
**Model 3**	NGT	Reference	Reference
	Pre-diabetes	1.08 (0.96–1.20)	1.07 (0.96–1.17)
	NDM	1.38 (1.20–1.57)	1.40 (1.21–1.59)
	KDM	1.96 (1.66–2.26)	2.07 (1.72–2.42)

Model 1 was adjusted for age.

Model 2 was adjusted for age, marital status, educational level and smoking status.

Model 3 was adjusted for all variables in model 2 plus BMI category, hypertension, hypercholesterolemia, CKD and history of CVD.

BMI: body mass index; CKD: chronic kidney disease; CVD: cardiovascular diseases; CI: confidence interval; NGT: normal glucose tolerance; NDM: newly diagnosed diabetes mellitus; KDM: known diabetes mellitus.

Supplementary Table 6 shows the adjusted rates per 1000 person-years (95% CI) of all-cause hospitalizations by glucose tolerance status from NB regression models. Based on models 1 and 2, the hospitalization rate was higher in pre-diabetes, NDM, and KDM than in NGT groups, in both men and women. According to model 3, after adjustment for all confounders, the adjusted rate of all-cause hospitalization in NDM and KDM participants was higher than NGT group, in both genders (Supplementary Table 6).

In Figure 1, we have shown the plot of adjusted rate of all-cause hospitalization per 1000 person-years over the age range by glucose tolerance status. Generally, the difference in hospitalization rates between the four groups increased with age, in both genders.

### Sensitivity analyses

The pattern of higher all-cause hospitalization rate per 1000 person-years with moving from NGT to KDM groups remained unchanged from NB models when we developed zero-inflated models.

The effect of glycemic control on hospitalization rate has been shown in Table 4. In the multivariate model, the incidence rate of hospitalization was 39% higher (1.39 (1.12–1.65) in poor controlled than fair controlled group.

**Table 4.** Estimates of incident rate ratios (IRRs) (95% CI) from negative binomial regression models by glycaemic control status, the TLGS study, 1999–2018.

Glycaemic control status	
Fair controlled (FP*G* < 10 mmol/l)	Reference
Poor controlled (FP*G* ≥ 10 mmol/l)	1.39 (1.12–1.65)*

The model was adjusted for age, sex, marital status, educational level, smoking status, BMI category, hypertension, hypercholesterolemia, CKD and history of CVD.

BMI: body mass index; CKD: chronic kidney disease; CVD: cardiovascular diseases; CI: confidence interval; FPG: fasting plasma glucose.

*p < 0.001.

When we limited follow-up time to 5 years after baseline, associations of glucose tolerance status with hospitalization risk remained statistically significant for pre-diabetes, NDM and KDM, compared with NGT group. After limiting follow-up time to 10 years, the associations were attenuated but remained significant for NDM and KDM categories (Supplementary Tables 7 and 8).

## Discussion

In this population-based study from the Middle East, we showed that in both genders, individuals with pre-diabetes, NDM, and KDM had a significantly higher rate of all-cause hospitalization during about two-decade follow-up than individuals with NGT status, after adjustment for age and demographic variables. Moreover, the higher risk of hospitalization for NDM and KDM survived after further adjustment for BMI category, HTN, hypercholesterolaemia, CKD, and prevalent CVD. Among those with T2DM, CHD, T2DM complications, and stroke events remained the most frequent hospitalization causes during the study period. Notably, among patients with KDM, the hospitalization rate was higher in patient with poor glycaemic control than those with fair glycaemic control.

In line with other studies, we generally confirmed that NDM and KDM were associated with a higher hospitalization rate. However, comparing our findings with other studies is not simple, due to different sources of data gathering (i.e. administrative registry data vs. population-based studies), various approaches to define glucose tolerance status, age at the recruitment time, study design (i.e. cross-sectional vs. cohort studies), duration of follow-up, level of confounding adjustment, and most importantly, insurance coverage for the study population.

A study in the USA, using ARIC study data, estimated the risk of hospitalization during 20 years of follow-up among individuals with pre-diabetes and diagnosed T2DM, and showed 3.1, 1.6 and 1.3 times higher risk for hospitalization in KDM, NDM and pre-diabetes groups, respectively, after adjustment for age, sex, race, and insurance status. In contrast to our data analysis, US men had a higher admission rate in all glucose tolerance status categories except for those with KDM and those with HbA1C ≥7% in which American women had higher admission rate [12]. An insurance registry-based study in Taiwan reviewed hospitalization trends in people with and without diabetes in 10 years. It showed that although the hospitalization rate decreased in patients with T2DM during a decade, they still had three times higher unadjusted hospitalization rates compared with those without T2DM [21]. An Spanish registry-based study showed a 2.5-fold increased age-adjusted hospitalization rate over the 14-year in those with diabetes that was more prominent among men [22]. A national administrative registry analysis in Italy showed that people with KDM had 2–3 times higher hospitalization rates after adjustment for age and sex [23].

We have previously shown that among T2DM patients, the risk for first CVD events was higher in women than men during seven years of follow-up [24]. In the current study with two-decades of follow-up, the risk of hospitalization was generally similar between genders among T2DM patients (including both KDM and NDM); however, the rate of CVD hospitalization (including first and recurrent events) due to CHD or stroke was 46% higher among men than women (1.46; 1.17–1.74). This higher rate of repeated hospitalization in men might be due to lower medical adherence after the first CVD event than women [25]. A population-based retrospective cohort in Canada, including 73700 individuals aged ≥25 years, reported higher CVD-related hospitalization and mortality rate among women with diabetes in comparison to their male counterparts [26]. A systematic review and meta-analysis of 64 cohorts, including 858000 individuals, demonstrated a 40% greater risk of first CHD in women with diabetes than men [27]. Another study in the US reported a higher rate of T2DM-related hospitalization in women than men, but age and sex interactions were found; the hospitalization rate was higher in women in the younger age group, and among men with older age [28].

We found that, among patients with KDM, individuals with poor controlled glucose (FPG ≥10 mmol/l) had a 43% higher rate of hospitalization, compared with their fair controlled counterparts. A multicentre study of 4700 participants in the United Kingdom showed a non-linear relationship between HbA1C level and the risk of hospitalization, with a significant increase in risk for HbA1C level above 7.7%. Moreover, for every 1% increase above the threshold, the risk for all-cause hospitalization raised 6.3% [29]. A retrospective cohort in New York involving 4700 patients with T2DM and heart failure (HF) showed an increased risk of hospitalization for HF only in those with HbA1C >9% (Hazard Ratio:1.2 (0.99–1.45)) [30]. When the effect of glycaemic control was evaluated in the ARIC study [12], KDM patients with HbA1C >7% had a 1.5 times higher hospitalization rate than those with HbA1C <7%. Using data from the Hong Kong diabetes registry, the relationship between glycaemic control and infectious disease hospitalization was examined; this study showed a 5–10% increase in hospitalization rate per 1% absolute increase in HbA1C, but the relation was not linear in all HbA1C levels. A U-shape relation between these two variables was found: a HbA1C level <6.0% and >8.0% were both associated with higher risk than HbA1C interval of 7.0–8.0%, after adjustment for demographic and clinical confounders [31].

In the present study, we showed that among T2DM patients, CHD, T2DM complications (including diabetic-nephropathy, diabetic foot ulcer, hypoglycaemia, diabetic ketoacidosis, hyperosmolar state), and stroke events, respectively, were the most common causes of hospitalization; whereas, neoplasms and infectious disease were in the lower ranks. In line with our study, CVD has been invariably the first leading cause of hospitalization in people with T2DM. A US study on 6500 participants in 2006, investigated common reasons for hospitalization in the urban population with diabetes and reported the diseases of the circulatory system, endocrine disease, and pneumonia as the most common causes [32]. The ARIC study [12], showed the same result as CVD, endocrine, and respiratory disease were the most common causes. The Spanish study found that CHF, neoplasm, and COPD were the leading causes of hospitalization in people with T2DM during 14 years of follow-up [22]. In Italy, a similar study demonstrated CVD, cerebrovascular disease, and T2DM-related eye complication as the most common causes [23].

Pre-diabetes as abnormal glucose tolerance status in our study was associated with a 13% increased rate of hospitalization; but after further adjustment for clinical confounders, the association not reached to statistically significance. The three most common causes for hospitalization in this group were CHD, neoplasm, and stroke events. Previous studies have shown an association between pre-diabetes and vascular complications and mortality. A meta-analysis on 129 prospective cohort studies in 2016 showed an increased risk of CVD (15%), CHD (16%), stroke (14%), and all-cause mortality (13%) in those with pre-diabetes in a 10-year follow-up [33]. Moreover, another meta-analysis in 2012 showed an increased risk of future stroke in individuals with pre-diabetes defined as having impaired fasting glucose (IFG) or impaired glucose tolerance (IGT) [34]. In some of these studies, the higher risk was eliminated after confounder adjustment, bolding the importance of other metabolic factors. We previously found that FPG ≥5.6 mmol/l was associated with CKD in men and HTN in women. Moreover, FPG ≥6.1 mmol/l and IGT were associated with stroke in men and CHD in women, respectively. We also recently showed that among participants with pre-diabetes at baseline, only those who developed diabetes had a higher risk of developing CVD/CHD [8]. In the current study, we did not consider the change of pre-diabetes to T2DM during this long-term follow-up. However, when we limited the duration of follow-up to 5 years, we unexpectedly found that pre-diabetes status still had significantly higher risk of hospitalization, compared with those with NGT group, even in the presence of a large set of confounders, suggesting that individuals with pre-diabetes are at high risk for hospitalization that is not fully explained by a later conversion to T2DM. In a recent study among patients with acute ischemic stroke, pre-diabetes was an independent predictor for early neurologic deterioration (OR = 2.02; 1.12–3.62) and in-hospital death (OR = 3.12; 1.06–9.09). On the other hand, diabetes was a significant independent factor for poor long-term outcomes (OR = 1.75; 1.09–2.78). The authors speculated that pre-diabetes population in the short term had lower usage of anti-platelet and statin medications than patients with diabetes [35].

As a strength, to our knowledge, this is the first study that examined the association between different levels of glucose intolerance using strict criteria with hospitalization during a two-decade follow-up in a region with high cardio-metabolic disorders [36]. Moreover, we precisely measured the confounders rather than relying on self-reported data. There are some limitations to consider: (1) we did not have data on the insurance status of the participants at the requirement and during follow-up; the factor strongly affected the hospitalization rate among patients. However, a statistical study in 2009 showed that 85% of Iranian families had at least one basic insurance; (2) we did not consider glucose tolerance and covariates changes during the follow-up; (3) we did not have data on the duration of diabetes; moreover, the level of HbA1C is not measured in TLGS cohort, due to the high cost for accurate and precise measurement of this important parameter by high-performance liquid chromatography (HPLC) method. Hence, we used FPG level as a surrogate of HbA1C to define the glycaemic control status; (4) In the TLGS, the urinary albumin assessment did not perform, therefore, this parameter was not considered in the CKD definition; (5) the current study was conducted in the metropolitan of Tehran, so our findings might not be extrapolated to the country’s rural zones; (6) we collected hospitalization data *via* annual telephone call. There was a possibility that someone couldn’t remember if they had a hospitalization. Moreover, the included participants was generally healthier than non-included participant. Therefore, our results may underestimate the true rates of hospitalization in our population.

In conclusion, in this community-based longitudinal study, we observed that individuals with pre-diabetes, NDM, and KDM had a significantly higher risk of hospitalization, compared with those with NGT. This effect became more prominent as the person aged. Generally, CVD was the leading cause of hospitalization among the population with diabetes, the issue that was more prominent among men. We found that in patients with KDM, fair glycaemic control significantly reduced the rate of hospitalization, which may potentially decrease the high economic burden of diabetes on the healthcare systems. Therefore, it is necessary to consider multicomponent policies aiming at slow down the increasing rate of pre-diabetes tsunamis as soon as possible [37] and implementing screening policies for early diagnosis of diabetes throughout the country.

## Supplementary Material

Supplemental Material

## Data Availability

The datasets used and/or analysed during the current study are available from the corresponding author on reasonable request.
