# Supplementary material for: The relationship between glucose intolerance status and risk of hospitalization during two decades of follow-up: Tehran lipid and glucose study
Source: Ann Med. 2022 Nov 16;54(1):3258–68. doi: 10.1080/07853890.2022.2143552 (PMC9673780; doi:10.1080/07853890.2022.2143552)
Supplement: Supplemental Material [file IANN_A_2143552_SM0674.docx]

9558 participants aged ≥30 years,

7927 recruited from Phase 1 (1999-2001)

, and 1631 recruited from Phase 2 (2002-2005)

**Exclusion:**

People with no follow-up data, n=879

Missing data on glucose tolerance status at baseline, n=398

Missing data on covariates at baseline, n=267

Final study sample, n=8014 (3638 men)

**Supplementary Figure1:** Flowchart of sample selection for the study, the TLGS study, 1999-2018

|  |
| --- |
| **Supplementary Figure 2.** The total number of all-cause hospitalizations by sex and baseline diabetes category over 18 years of follow-up, The Tehran lipid and glucose study, 1999-2018 |

| **Supplementary Table 1:** Categories of hospitalization causes by ICD-10 codes | | | | | | | | |
| --- | --- | --- | --- | --- | --- | --- | --- | --- |
| **CHD** | **Stroke** | **Infectious disease** | **Respiratory disease** | **DM complication** | **HTN complication** | **Neoplasm** | **Trauma** | **Others** |
| Fatal MI  I22 | Fatal stroke  I64 | Sepsis  A40-41 | Asthma  J45-46 | Diabetic nephropathy  E11.2 | Malignant HTN  I10.10 | Breast cancer  C50 | Accident  V01-99 | Benign tumors  D10-36 |
| Non-fatal MI  I21 | Non-fatal stroke  I63 | Pneumonia  J12-18 | Chronic bronchitis  J40-42 | Diabetic foot ulcer  E11.5 | HTN-related cardiac disease  I11 | Lung cancer  C34 | Fractures  T12-14.2 | Un-classified non-coronary disease  I30-52 |
| Definite CHD  I25 |  |  | Emphysema  J43 | Hypoglycemia  E16.0,1,2 | HTN crisis  I16.0,1,9 | Gastrointestinal cancer  C15-21,23-24,26 |  | Poisoning  T51-65 |
| Sudden cardiac death  I46 |  |  | Bronchiectasis  J47 | Diabetic ketoacidosis  E11.1 | HTN complication  I12-13 | Prostate cancer  C61 |  | Drug overdoses  T36-50 |
| Heart failure  I50 |  |  | COPD  J44 | Hyperosmolar state  E11.00 | HTN in pregnancy  O.13 | Bladder cancer  C67 |  | Unclassified disease |
| Unstable angina  I20 |  |  |  | Other DM complication  E11.3,4,6,7,8 |  | Uterus cancer  C54-55 |  |  |
| Peripheral artery disease  I73 |  |  |  | DM metabolic control  E70-90 |  | Liver cancer  C22 |  |  |
|  |  |  |  | GDM  O.24 |  | Pancreas cancer  C25 |  |  |
|  |  |  |  | Metabolic surgery for patients with diabetes  Z.98 |  | Other cancers  C00-97(excluding above) |  |  |
| **ICD-10;** International Classification of Diseases 10th Revision; **CHD**: coronary heart disease; **MI**: myocardial infarction; **COPD**: Chronic obstructive pulmonary disease; **DM**: diabetes mellitus; **GDM**; gestational diabetes mellitus; **HTN**: hypertension | | | | | | | | |

| **Supplementary Table 2:** Baseline characteristics of included and non-included participants: the TLGS study, 1999-2018 | | | | | |
| --- | --- | --- | --- | --- | --- |
|  |  | | Included participants  (n=4014) | Non-included participants  (n=1544) | P-value |
| **Continuous variables** | | |  |  |  |
|  | Age (years) | | 47.4 (12.3) | 48.2 (13.9) | 0.028 |
|  | BMI (kg/m^2^) | | 27.5 (4.5) | 27.2 (4.6) | 0.024 |
|  | SBP (mmHg) | | 121.9 (20.1) | 122.6 (20.9) | 0.210 |
|  | DBP (mmHg) | | 78.7 (11.1) | 78.8 (10.9) | 0.668 |
|  | FPG (mmol/L) | | 5.6 (1.9) | 5.7 (2.2) | 0.641 |
|  | TC (mmol/L) | | 5.5 (1.1) | 5.6 (1.2) | 0.317 |
|  | TG (mmol/L) | | 2.1 (1.3) | 2.1 (1.4) | 0.802 |
|  | e-GFR (mL/min per 1.73 m^2^) | | 71.8 (13.5) | 70.6 (14.9) | 0.004 |
| **Categorical variables** | | |  |  |  |
|  | Smoking (%) | |  |  |  |
|  |  | Never | 5956 (74.3) | 946 (70.4) | 0.001 |
|  |  | Past | 719 (9.0) | 116 (8.6) |  |
|  |  | Current | 1339 (16.7) | 282 (21.0) |  |
|  | Marital status (%) | |  |  |  |
|  |  | Single | 311 (3.9) | 61 (4.0) | 0.671 |
|  |  | Married | 7098 (88.6) | 1351 (87.8) |  |
|  |  | Widowed/divorced | 605 (7.5) | 126 (8.2) |  |
|  | Educational level (%) | |  |  |  |
|  |  | <6 years | 3220 (40.2) | 651 (42.7) | 0.174 |
|  |  | 6-12 | 3838 (47.9) | 697 (45.8) |  |
|  |  | ≥12 | 956 (11.9) | 175 (11.5) |  |
|  | Prevalent CVD (yes) | | 477 (6.0) | 125 (8.1) | 0.002 |
|  | Hypertension (yes) | | 2160 (27.0) | 372 (27.9) | 0.464 |
|  | CKD (yes) | | 1469 (18.3) | 291 (23.1) | <0.001 |
|  | Hypercholesterolemia (yes) | | 4843 (60.4) | 806 (52.2) | <0.001 |
|  | BMI status (%) | |  |  |  |
|  |  | Normal | 2380 (29.7) | 398 (31.9) | 0.128 |
|  |  | Overweight | 3482 (43.4) | 544 (43.6) |  |
|  |  | Obese | 2152 (26.9) | 305 (24.5) |  |
| Values are shown as mean (standard deviation) for continuous variables and frequency (percent) for categorical variables.  **BMI**: body mass index; **SBP**: systolic blood pressure; **DBP**: diastolic blood pressure; **FPG**: fasting plasma glucose; **2 h-PLPG:** 2-h post load plasma glucose; **TC**: total cholesterol; **TG**: triglycerides; **e-GFR**: estimated glomerular filtration rate; **CKD**: chronic kidney disease; **CVD**: cardiovascular diseases | | | | | |

| **Supplementary Table 3:** Total number of all-cause and cause specific hospitalization in men by baseline glucose tolerance status: the TLGS study, 1999-2018 | | | | |
| --- | --- | --- | --- | --- |
|  | Normal  (n=2311) | Pre-diabetes  (n=836) | Newly-diagnosed diabetes  (n=343) | Known diabetes  (n=148) |
|  | Number | Number | Number | Number |
| **All cause hospitalizations** | 2228 | 1105 | 655 | 347 |
| **CHD** | 702 | 358 | 300 | 130 |
| **Stroke** | 71 | 62 | 50 | 36 |
| **Neoplasm** | 181 | 82 | 41 | 17 |
| **HTN** | 29 | 7 | 4 | 5 |
| **T2DM complications** | 7 | 17 | 53 | 63 |
| Infectious disease | 72 | 39 | 30 | 21 |
| Respiratory disease | 57 | 14 | 1 | 1 |
| **Traumatic** | 131 | 43 | 16 | 9 |
| **Others** | 978 | 483 | 160 | 65 |
| **CHD**: coronary heart disease; **MI**: myocardial infarction; **COPD**: Chronic obstructive pulmonary disease; T2DM: diabetes mellitus;  **GDM**; gestational diabetes mellitus; **HTN**: hypertension | | | | |
|  | | | | |

| **Supplementary Table 4:** Number of all-cause and cause specific hospitalization in women by baseline diabetes status category: the TLGS study, 1999-2018 | | | | |
| --- | --- | --- | --- | --- |
|  | Normal  (n=2683) | Pre-diabetes  (n=1025) | Newly-diagnosed diabetes  (n=407) | Known diabetes  (n=261) |
|  | Number | Number | Number | Number |
| **All cause hospitalizations** | 2583 | 1323 | 738 | 672 |
| **CHD** | 381 | 298 | 229 | 251 |
| **Stroke** | 66 | 39 | 28 | 23 |
| **Neoplasm** | 220 | 110 | 30 | 24 |
| **HTN** | 56 | 45 | 25 | 16 |
| **T2DM complications** | 18 | 25 | 68 | 130 |
| Infectious disease | 66 | 34 | 29 | 38 |
| Respiratory disease | 17 | 18 | 4 | 2 |
| **Traumatic** | 115 | 45 | 26 | 18 |
| **Others** | 1644 | 709 | 299 | 170 |
| **CHD**: coronary heart disease; **MI**: myocardial infarction; **COPD**: Chronic obstructive pulmonary disease; T2DM: diabetes mellitus;  **GDM**; gestational diabetes mellitus; **HTN**: hypertension | | | | |

| **Supplementary Table 5:** Crude rates of all-cause and cause specific hospitalization per 1000 person-years in total population by baseline glucose tolerance status: the TLGS study, 1999-2018 | | | | | | | | |
| --- | --- | --- | --- | --- | --- | --- | --- | --- |
|  | Normal  n=4994)) | | Pre-diabetes  n=1861)) | | Newly-diagnosed diabetes  ((n=750 | | Known diabetes  n=409)) | |
|  | Number | Crude rate | Number | Crude rate | Number | Crude rate | Number | Crude rate |
| **All cause hospitalizations** | 4811 | 62.6 (59.9-65.3) | 2428 | 88.1 (82.5-93.8) | 1393 | 134.4 (122.8-146.1) | 1019 | 223.3 (199.3-247.3) |
| **CHD** | 1083 | 14.2 (13.1-15.4) | 656 | 23.8 (21.1-26.5) | 529 | 51.8 (44.6-59.1) | 381 | 83.5 (69.4-97.4) |
| **Stroke** | 137 | 1.8 (1.4-2.1) | 101 | 3.8 (2.8-4.7) | 78 | 7.6 (5.6-9.5) | 59 | 15.1 (9.8-20.2) |
| **Neoplasm** | 401 | 6.1 (5.1-7.1) | 192 | 8.0 (6.1-9.9) | 71 | 7.7 (4.9-10.5) | 41 | 10.1 (4.9-15.3) |
| **HTN** | 85 | 1.1 (0.7-1.3) | 52 | 1.8 (1.2-2.3) | 29 | 2.6 (1.4-3.8) | 21 | 4.3 (2.2-6.3) |
| **T2DM complications** | 25 | 0.3 (0.1-0.4) | 42 | 1.4 (0.9-1.9) | 121 | 11.1 (8.3-13.7) | 193 | 40.7 (32.5-49.0) |
| Infectious disease | 138 | 1.7 (1.4-2.1) | 73 | 2.6 (1.9-3.3) | 59 | 5.9 (3.7-8.1) | 59 | 11.9 (8.8-15.1) |
| Respiratory disease | 74 | 1.1 (0.3-1.9) | 32 | 1.2 (0.6-1.7) | 5 | 0.4 (0.01-1.06) | 3 | 0.6 (0.01-1.2) |
| **Traumatic** | 246 | 3.1 (2.6-3.5) | 88 | 3.0 (2.3-3.7) | 42 | 3.8 (2.5-5.1) | 27 | 5.4 (3.3-7.5) |
| **Others** | 2622 | 33.1 (31.5-34.7) | 1192 | 41.5 (38.4-44.7) | 459 | 42.3 (37.0-47.6) | 235 | 47.8 (39.2-56.3) |
| **CHD**: coronary heart disease; **MI**: myocardial infarction; **COPD**: Chronic obstructive pulmonary disease; T2DM: diabetes mellitus;  **GDM**; gestational diabetes mellitus; **HTN**: hypertension | | | | | | | | |

| **Supplementary Table 6:** Adjusted rates of all-cause hospitalization per 1,000 person-years (95% CI) by baseline diabetes status from negative binomial models: the TLGS study, 1999–2018 | | | |
| --- | --- | --- | --- |
|  | Diabetes status | Men | Women |
| Model 1 | NGT | 62.4 (59.0-65.9) | 62.6 (59.3-65.9) |
|  | Pre-diabetes | 71.0 (65.1-77.3) | 70.9 (65.4-76.8) |
|  | NDM | 98.1 (86.8-110.9) | 95.5 (84.8-107.4) |
|  | KDM | 138.0 (115.5-164.8) | 145.7 (126.6-167.7) |
|  |  |  |  |
| Model 2 | NGT | 59.5 (53.9-65.7) | 65.1 (60.4-70.3) |
|  | Pre-diabetes | 68.2 (60.8-76.6) | 73.2 (66.6-80.4) |
|  | NDM | 92.9 (80.3-107.5) | 98.1 (86.3-111.4) |
|  | KDM | 132.0 (108.8-160.2) | 149.8 (129.1-173.9) |
|  |  |  |  |
| Model 3 | NGT | 50.6 (44.8-57.1) | 50.2 (44.5-56.7) |
|  | Pre-diabetes | 55.0 (47.6-63.6) | 53.8 (46.6-62.0) |
|  | NDM | 70.1 (58.9-83.5) | 70.6 (59.7-83.6) |
|  | KDM | 99.4 (80.7-122.4) | 104.2 (86.9-124.9) |
| Model 1 was adjusted for age  Model 2 was adjusted for age, marital status, educational level and smoking status  Model 3 was adjusted for all variables in model 2 plus BMI category, hypertension, hypercholesterolemia, CKD and history of CVD  **BMI**: body mass index; **CKD**: chronic kidney disease; **CVD**: cardiovascular diseases; **CI:** confidence interval; **NGT:** normal glucose tolerance; **NDM**: newly diagnosed diabetes mellitus; **KDM**: known diabetes mellitus | | | |

| **Supplementary Table 7:** Estimates of incident rate ratios (IRRs) (95% CI) from negative binomial regression models in men and women, limiting follow-up time to 5 years after baseline, the TLGS study, 1999-2018 | | | |
| --- | --- | --- | --- |
|  | **Diabetes status** | **Men** | **Women** |
| **Model 1** | Normal | Reference | Reference |
|  | Pre-diabetes | 1.35 (1.02-1.67) | 1.46 (1.08-1.83) |
|  | NDM | 1.82 (1.26-2.38) | 1.88 (1.31-2.45) |
|  | KDM | 2.67 (1.83-3.51) | 3.37 (2.38-4.36) |
|  |  |  |  |
| **Model 2** | Normal | Reference | Reference |
|  | Pre-diabetes | 1.39 (1.05-1.72) | 1.48 (1.10-1.86) |
|  | NDM | 1.82 (1.27-2.38) | 1.90 (1.33-2.48) |
|  | KDM | 2.79 (1.92-3.65) | 3.39 (2.39-4.39) |
|  |  |  |  |
| **Model 3** | Normal | Reference | Reference |
|  | Pre-diabetes | 1.35 (1.02-1.67) | 1.41 (1.06-1.76) |
|  | NDM | 1.66 (1.15-2.17) | 1.77 (1.24-2.30) |
|  | KDM | 2.40 (1.66-3.15) | 2.98 (2.07-3.90) |
| Model 1 was adjusted for age  Model 2 was adjusted for age, marital status, educational level and smoking status  Model 3 was adjusted for all variables in model 2 plus BMI category, hypertension, hypercholesterolemia, CKD and history of CVD  **BMI**: body mass index; **CKD**: chronic kidney disease; **CVD**: cardiovascular diseases; **CI:** confidence interval; **NGT:** normal glucose tolerance; **NDM**: newly diagnosed diabetes mellitus; **KDM**: known diabetes mellitus | | | |

| **Supplementary Table 8:** Estimates of incident rate ratios (IRRs) (95% CI) from negative binomial regression models in men and women, limiting follow-up time to 10 years after baseline, the TLGS study, 1999-2018 | | | |
| --- | --- | --- | --- |
|  | **Diabetes status** | **Men** | **Women** |
| **Model 1** | Normal | Reference | Reference |
|  | Pre-diabetes | 1.06 (0.90-1.23) | 1.14 (0.98-1.31) |
|  | NDM | 1.62 (1.31-1.93) | 1.59 (1.30-1.89) |
|  | KDM | 1.88 (1.45-2.31) | 2.38 (1.88-2.88) |
|  |  |  |  |
| **Model 2** | Normal | Reference | Reference |
|  | Pre-diabetes | 1.08 (0.91-1.24) | 1.14 (0.97-1.31) |
|  | NDM | 1.61 (1.31-1.91) | 1.58 (1.29-1.87) |
|  | KDM | 1.90 (1.48-2.32) | 2.37 (1.87-2.87) |
|  |  |  |  |
| **Model 3** | Normal | Reference | Reference |
|  | Pre-diabetes | 1.03 (0.87-1.18) | 1.09 (0.94-1.25) |
|  | NDM | 1.43 (1.17-1.68) | 1.48 (1.21-1.74) |
|  | KDM | 1.65 (1.30-1.99) | 2.15 (1.69-2.62) |
| Model 1 was adjusted for age  Model 2 was adjusted for age, marital status, educational level and smoking status  Model 3 was adjusted for all variables in model 2 plus BMI category, hypertension, hypercholesterolemia, CKD and history of CVD  **BMI**: body mass index; **CKD**: chronic kidney disease; **CVD**: cardiovascular diseases; **CI:** confidence interval; **NGT:** normal glucose tolerance; **NDM**: newly diagnosed diabetes mellitus; **KDM**: known diabetes mellitus | | | |
